# Primary anesthesia provider characteristics and risk factors for intraoperative medication errors: a retrospective cohort study

**DOI:** 10.1186/s12871-025-03539-4

**Published:** 2025-12-13

**Authors:** Kohei Ikeda, Masayoshi Koike, Seirin Yamazaki, Sae Nakamura, Shoichi Uezono

**Affiliations:** Department of Anesthesiology, The Jikei University School of Medicine, 3- 19-18, Nishi-Shimbashi Minato-ku, Tokyo, Japan

**Keywords:** Intraoperative medication errors, Anesthesia safety, Firth’s penalized logistic regression, Patient safety, Retrospective cohort study, Anesthesia provider experience

## Abstract

**Background:**

Intraoperative medication errors, although uncommon, can result in considerable patient harm. Evidence remains limited regarding anesthesia provider-level and perioperative risk factors. This study aimed to evaluate whether anesthesia provider characteristics—particularly experience level and team composition—are statistically associated with intraoperative medication errors after multivariable adjustment.

**Methods:**

We conducted a retrospective observational study of 100,093 surgical cases managed under anesthesia at a university-affiliated tertiary hospital in Japan between August 2011 and December 2023. Data were extracted from an electronic anesthesia record system linked to institutional medical records. Medication errors were mainly identified through anesthesia provider self-reporting, supplemented by reports from operating room nurses. Predictor variables included patient characteristics, procedural details, and the main explanatory variable was anesthesia provider configuration: attending, and resident or intern under the supervision of an attending anesthesiologist. Firth’s penalized logistic regression was used to adjust for confounding variables identified via a directed acyclic graph.

**Results:**

Intraoperative medication errors occurred in 102 of 100,093 procedures (0.10%). Compared with attending anesthesiologists, the odds of medication error—adjusted for the familywise error rate (FWER) using the Holm–Bonferroni method—were significantly higher when care involved residents [OR 2.713; 95% CI, 1.283–6.815; *P* = 0.007] or interns [OR 3.272; 95% CI, 1.508–8.368; *P* = 0.003]. After multiplicity adjustment, no other factors—including age, American Society of Anesthesiologists Physical Status (ASA-PS) classification, and surgical urgency—were statistically associated with error risk. Sensitivity analyses confirmed the robustness of the main findings across different covariate sets.

**Conclusions:**

Anesthesia provider characteristics were statistically associated with intraoperative medication error risk. These findings suggest the need for strengthened supervision, structured team roles, and systems-based safeguards in perioperative medication safety.

**Supplementary Information:**

The online version contains supplementary material available at 10.1186/s12871-025-03539-4.

## Introduction

Perioperative medication errors remain one of the most critical challenges in modern healthcare [1]. The operating room presents a particularly high-risk setting, where clinicians must manage complex workflows and multiple medications under considerable time pressure and cognitive stress [2, 3]. These conditions create a setting especially prone to errors, potentially leading to significant morbidity and mortality [4].

Several large-scale studies have provided insights into the incidence of medication errors in surgical settings. Llewellyn et al. reported 111 medication errors among 30,412 surgical cases (0.37%) [5], while Cooper et al. identified 52 errors in 10,574 procedures (0.49%) [2]. A more recent study by Zhang et al. found a higher rate of 179 errors in 24,380 cases (0.73%) [6]. However, the true incidence may be underestimated due to potential underreporting in retrospective analyses. Wahr et al., through a comprehensive analysis, identified key contributing factors such as communication breakdowns among team members, environmental stressors, provider fatigue, and the complexity of coordinating multiple healthcare providers during perioperative care [7]. Their expert consensus also emphasized the importance of provider experience—particularly among anesthesiologists and anesthesia residents. However, the exact relationship between provider experience and error risk remains unclear.

Despite multiple safety checks, medication errors persist, indicating deeper system issues such as communication breakdowns, inefficient workflows, and inadequate staffing; Katz et al. highlighted major barriers to prevention and accurate reporting of perioperative incidents [8]. Involvement of multiple anesthesia providers may increase medication-administration complexity and error risk. Evidence specific to intraoperative medication errors—especially provider characteristics, procedural variables, and team dynamics—remains limited [9, 10]. Growing evidence nonetheless implicates provider factors—experience, cognitive workload, and psychological stress—in medication safety within increasingly complex anesthesia practice and healthcare systems [2, 11–14], yet few studies have evaluated these relationships with robust causal methods. To address this gap, we applied a directed acyclic graph (DAG) informed multivariable modeling and Firth’s penalized logistic regression to a large single-center dataset (>100,000 cases) to estimate the independent effect of provider characteristics on error risk, hypothesizing that less experience and multi-provider involvement would increase intraoperative medication errors after adjustment for patient-, procedural-, and contextual factors.

## Materials and methods

### Study population

We conducted a single-center retrospective observational study at a university-affiliated tertiary hospital in Japan (August 2011–December 2023). The Ethics Committee of The Jikei University School of Medicine for Biomedical Research approved the protocol (36–143[12252]). All surgical cases managed by the Department of Anesthesiology were included. We excluded minor procedures (e.g., central venous catheterization), cases entering the operating room without surgery, cases primarily managed by non-anesthesiologists, and records with missing covariates. Owing to the retrospective design, informed consent was waived; study details were posted online with an opt-out option.

#### Definition of medication error

Medication errors were defined as preventable events leading to inappropriate medication use or patient harm while under a healthcare professional’s control. All intraoperative medication errors were initially identified through self-reports by anesthesia providers or reports from operating room nurses. To ensure consistent case identification and reduce detection bias, two attending anesthesiologists independently reviewed each reported event to confirm that it met the predefined study criteria for intraoperative medication error. Events that occurred before patient entry into the operating room, reflected documentation-only mistakes, or involved incorrect preparation not actually administered to the patient were excluded. Error types followed Webster et al. [15]: omission, repetition, substitution, insertion, incorrect dose, and incorrect route/site. This scheme is widely used in anesthesia safety research. Severity was graded using the incident severity framework approved by the Medical Safety Management Council of National University Hospitals in Japan (Supplementary Table 1).

### Data collection and covariate selection

Medication errors were identified from anesthesia-provider self-reports and nurse-initiated reports submitted via the hospital incident-reporting system or the intraoperative electronic anesthesia record (ORSYS, Philips, Japan). The patient-safety department reconciled duplicates, and we manually verified each case to ensure inclusion of unique events only. Independent covariates were prespecified for clinical relevance and prior evidence [5, 16, 17]. Data were extracted from the electronic anesthesia record and hospital charts; cases with missing covariates were excluded. Variables collected were:

- Patient: age, sex.
- Surgical-related factors: department (17 specialties plus “others”), urgency (elective/emergency).
- Anesthesia-related factors: American Society of Anesthesiologists Physical Status (ASA-PS I = healthy, II = mild systemic disease, III = severe systemic disease, IV = life-threatening disease); anesthesia type (volatile general anesthesia, total intravenous anesthesia [TIVA], regional anesthesia, monitored anesthesia care [MAC]); anesthesia duration; primary provider; start time; day of surgery.
- Medication error: occurrence and context.

Primary provider categories were attending anesthesiologist alone, intern with attending supervision, and resident with attending supervision. In Japan, interns are PGY1–2, residents PGY3–5, and attendings ≥ PGY6; one attending may supervise 1–3 trainees. “Attending alone” denotes no trainee involvement. Start time was classified as day (06:00–18:00) vs. night (18:00–06:00), and day of surgery as weekday vs. holiday. To avoid collinearity, operation duration was excluded in favor of anesthesia duration, which better reflects medication exposure. Missingness was minimal (6 cases; 0.006%); assuming missing completely at random, we performed complete-case analysis without multiple imputation. Data on supervision intensity, intraoperative handoffs, and provider workload were not available with sufficient granularity and were therefore not included in the adjustment sets. During the study period, our institution maintained standardized manual medication verification procedures. Before surgical incision, perioperative antibiotics were confirmed by three personnel—the scrub nurse, anesthesiologist, and surgeon—to ensure correct drug, dose, and timing. All anesthetic syringes were labeled with color-coded, drug-specific labels. To prevent route errors, syringes for neuraxial anesthesia were of a distinct design and connector type, incompatible with intravenous systems. Bar-code scanning or electronic double-check systems were not implemented during the study period.

#### Sample size and power

Because the cohort size was fixed, we justified study size by the minimum detectable effect. Using the observed exposure distribution (any trainee involvement vs. attending-only; ~85% vs. 15%) and a baseline error risk of ~ 0.039% in the attending-only group (estimated from the observed distribution of errors across exposure strata), we approximated power with a two-sample comparison of proportions under the rare-event assumption (two-sided α = 0.05). The available sample affords ~ 80% power to detect an odds ratio (OR) ≥ 1.8 for the primary contrast.

#### Statistical analysis

Given the rare outcome (~ 0.1%), conventional logistic regression risks small-sample bias and separation. We therefore applied Firth’s penalized likelihood logistic regression (logistf v1.24.1) in R (v4.5.0; R Foundation for Statistical Computing, Vienna, Austria). The primary exposure—primary anesthesia provider (attending; resident with supervision; intern with supervision)—was included in all models. Covariates were prespecified using DAG informed by clinical knowledge and prior literature [18, 19]. The minimal sufficient adjustment set comprised age, ASA-PS, surgical department, and urgency (elective/emergency) (Supplementary Fig. 1 A, B); these were entered simultaneously in the main model. Putative mediators/colliders (e.g., anesthesia type, anesthesia duration) were excluded from the primary model and assessed in sensitivity analyses. The DAG was built in DAGitty (version 3.1, http://www.dagitty.net). Because multiple contrasts were evaluated, we controlled familywise error rate (FWER) using the Holm–Bonferroni procedure and the false discovery rate (FDR) using the Benjamini–Hochberg procedure. To avoid overstating null findings, statements of ‘no significant association’ were limited to contrasts with narrow multiplicity-adjusted confidence intervals excluding clinically relevant effect sizes.

### Model fit evaluation

Model performance was assessed using the area under the receiver operating characteristic curve (AUC), calibration plots, and the Brier score. The AUC was used to evaluate the model’s discriminative ability. Calibration was visually assessed by comparing predicted and observed event probabilities using a smoothed calibration curve. The Brier score was calculated as a measure of overall accuracy, reflecting both discrimination and calibration.

### Sensitivity analyses

To assess the robustness of our findings, we conducted two sensitivity analyses:

1. A full Firth’s penalized logistic regression model including all available covariates (both categorical and continuous), to evaluate the potential influence of additional factors.
2. A Firth’s penalized logistic regression model using an alternative DAG-based minimal adjustment set (age, surgical department, surgical urgency, and type of anesthesia; see Supplementary Fig. 1 C), to test the robustness of the primary results.

## Results

### Cohort summary and incidence of intraoperative medication errors

**Fig. 1:**
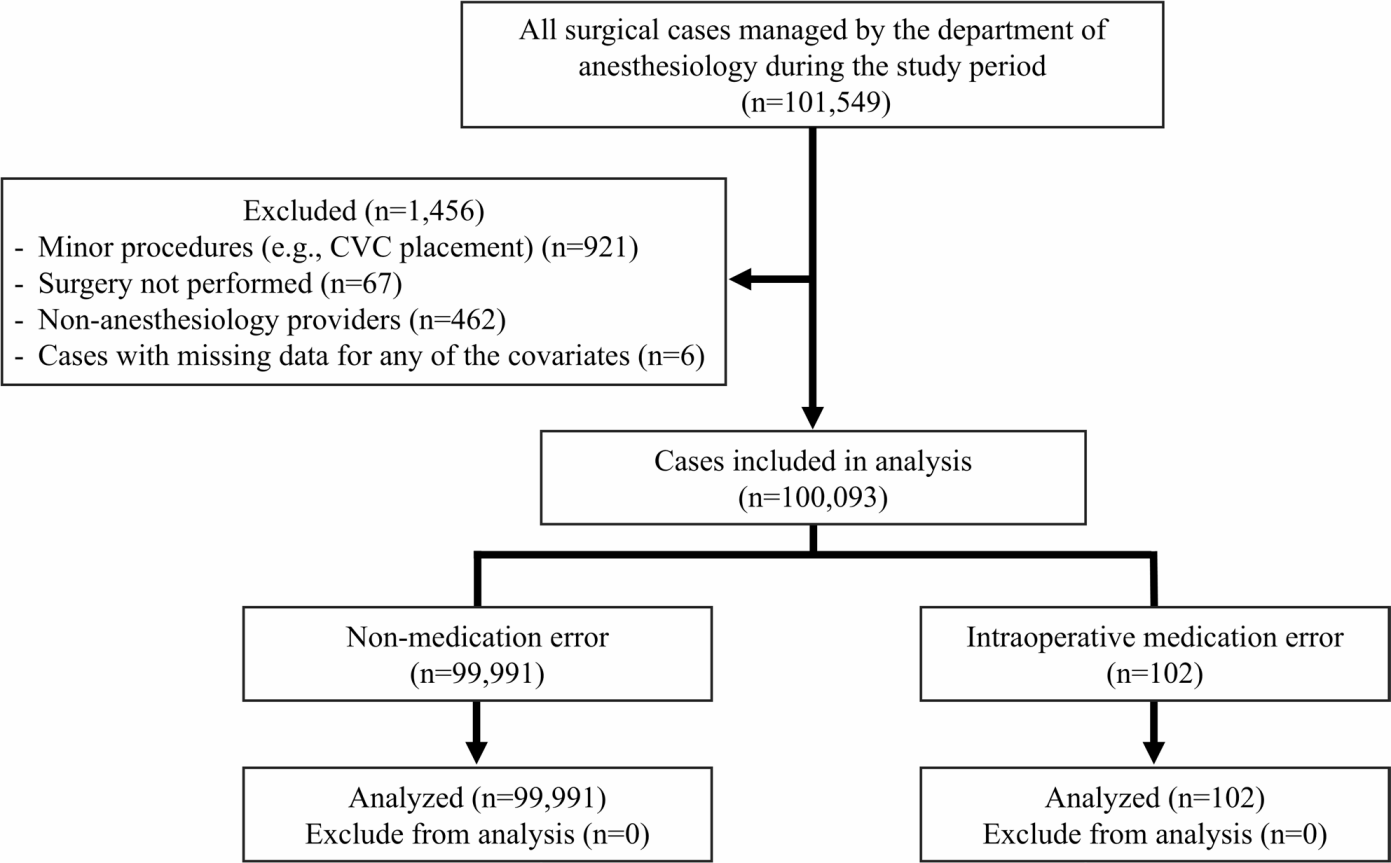
Flow diagram illustrating case selection and exclusion criteria. Of 101,549 surgical cases managed by the Department of Anesthesiology during the study period, 1,456 were excluded due to minor procedures such as central venous catheterization (*n* = 921), aborted surgery (*n* = 67), non-anesthesiology primary providers (*n* = 462), or missing data for covariates (*n* = 6). A total of 100,093 cases were included in the final analysis, comprising 102 cases with intraoperative medication errors and 99,991 without errors. CVC, central venous catheterization

As shown in Fig. 1, a total of 101,549 surgical procedures were managed by the department of anesthesiology during the study period. Of these, 1,456 cases were excluded based on predefined criteria, leaving 100,093 cases for final analysis. A total of 107 intraoperative medication-error events were initially reported through self-reports by anesthesia providers or reports from operating room nurses. After independent verification by two attending anesthesiologists, five events were excluded as not meeting the study definition of intraoperative medication error (e.g., preoperative administration errors or unadministered dosage adjustments), resulting in 102 confirmed events analyzed. Overall, 102 intraoperative medication errors (0.102%) were identified. The most common error types were incorrect dose (31.4%), substitution (21.6%), and omission (17.6%), followed by insertion (15.7%), repetition (7.8%), and incorrect route/site (5.9%) (Table 1). Antibiotics were the most frequently involved drug class, with remifentanil being the most commonly omitted medication. Among the intraoperative medication errors identified, 10 cases (9.8%) were attributable to communication failures, frequently occurring during transitions of care such as intraoperative handoffs. Omission errors mainly involved remifentanil and antibiotics. Most represented temporary interruptions or delays in restarting continuous infusions (e.g., after syringe or line changes) or omitted preoperative antibiotic doses according to protocol schedules. Rare omissions of sevoflurane or succinylcholine occurred when the anesthetic plan was changed intraoperatively (e.g., conversion from volatile to TIVA anesthesia or substitution of rocuronium during rapid-sequence induction). Incorrect-dose errors reflected deviations from institutional or weight-based dosing protocols—such as fixed 1000 mg acetaminophen or antibiotic doses administered to patients of low body weight—rather than toxic overdoses. All local anesthetic doses (e.g., levobupivacaine) remained below established maximum safe limits.

**Table 1:** Classification of medication error types and involved drugs. Types of intraoperative medication errors were classified into six categories: incorrect dose, substitution, omission, insertion, repetition, and incorrect route or site. The number and percentage of each error type are shown, along with representative drugs and their frequency

Type of errors	*n*, (%)	Drugs (frequency)
Incorrect dose	32 (31.4)	antibiotics (7), fentanyl (4), levobupivacaine (4), morphine (3), rocuronium (3), acetaminophen (3), remifentanil (2), dexmedetomidine (1), amiodarone (1), ephedrine (1), phenylephrine (1), lidocaine (1),
Substitution	22 (21.6)	atropine (4), nicardipine (3), antibiotics (2), rocuronium (2), protamine (2), midazolam (1), ketamine (1), vasopressin (1), thiamylal (1), sugammadex (1), adrenaline (1), noradrenaline (1), ephedrine (1), phenylephrine (1)
Omission	18 (17.6)	remifentanil (10), propofol (3), antibiotics (2), sevoflurane (1), succinylcholine (1), levobupivacaine (1)
Insertion	16 (15.7)	acetaminophen (5), rocuronium (4), antibiotics (2), propofol (1), sugammadex (1), neostigmine (1), protamine (1), steroid (1)
Repetition	8 (7.8)	antibiotics (5), steroid (1), fentanyl (1), acetaminophen (1)
Incorrect route/site	6 (5.9)	ropivacaine (2), levobupivacaine (2), vasopressin (1), propofol + fentanyl + rocuronium (1)

### Distribution and severity of medication errors by anesthesia provider type

Table 2 presents the distribution of medication errors by anesthesia provider type and severity level. Residents were responsible for the highest proportion of errors (46.1%), followed by interns (37.3%) and attending anesthesiologists (16.6%). Most errors were of lower severity (Level 1 or 2). However, Level 3a events occurred in 6 cases involving residents, 5 involving interns, and 2 involving attending anesthesiologists. Notably, all four Level 3b events—requiring intensive care—occurred in cases managed by residents (Supplementary Table 3). These incidents caused only temporary harm requiring intervention, and all affected patients fully recovered without permanent sequelae. No permanent injuries (Level 4) or deaths (Level 5) were directly attributable to medication errors in our cohort.

**Table 2:** Distribution of medication errors by responsible anesthesia provider and incident severity level. The table presents the number and percentage of intraoperative medication errors according to the role of the responsible anesthesia provider (intern, resident, or attending anesthesiologist) who administered the incorrect medication. Incident severity was classified based on the Japanese National system, ranging from level 1 (no harm) to level 3b (temporary harm requiring intervention)

Anesthesia provider responsible for medication error	Number of errors, *n* (%)	Level 1 (*n* = 47)	Level 2 (*n* = 38)	Level 3a (*n* = 13)	Level 3b (*n* = 4)
Resident	47 (46.1)	21	16	6	4
Intern	38 (37.3)	18	15	5	0
Attending anesthesiologist	17 (16.6)	8	7	2	0

### Baseline characteristics comparison between groups

Baseline characteristics showed no significant differences between error and non-error groups for patient demographics (age, sex), surgical factors (department, ASA-PS, anesthesia type, urgency), or timing variables (day/night, weekday/holiday) (Table 3). However, anesthesia provider type differed significantly (*P* = 0.029): errors occurred less frequently with management by attending anesthesiologist (non-error; 15.4% vs. error; 5.9%) compared with resident + attending anesthesiologist (non-error; 46.3% vs. error; 51.0%) or intern + attending anesthesiologist (non-error; 38.3% vs. error; 43.1%). Cases with errors had significantly longer anesthesia durations (251 ± 152 vs. 216 ± 141 min, *P* = 0.012). This difference in anesthesia duration reflects an unadjusted, descriptive comparison. Anesthesia duration was not included in the multivariable model because, according to the DAG, it lies on a downstream path between the primary provider and medication error and therefore functions as a mediator/collider, not a confounder. Its exclusion prevents introduction of collider bias in estimating the total effect.

**Table 3:** Baseline characteristics of surgical cases with and without intraoperative medication errors. Continuous variables are presented as mean (standard deviation, SD), and categorical variables as counts and percentages. ASA-PS, American society of anesthesiologists physical Status; TIVA, total intravenous anesthesia; MAC, monitored anesthesia care

Variables	Non- Medication Error (*n* = 99,991)	Medication Error (*n* = 102)	*P* value
Age, years (SD)	50 (23)	52 (20)	0.326
Sex, n (%)			
Male	51,047 (51.1)	46 (45.1)	0.236
Female	48,944 (48.9)	56 (54.9)	
Department of surgery, n (%)			
Otorhinolaryngology Orthopedic Obstetrics and Gynecology Gastrointestinal Neurosurgery Plastic Urology Pediatric Hepato-Biliary-Pancreatic Vascular Breast and Endocrine Thoracic Cardiac Ophthalmology Dermatology Psychiatry Dental and Oral Other Internal Medicine	19,152 (19.2) 13,282 (13.3) 12,358 (12.4) 8808 (8.8) 7535 (7.5) 6721 (6.7) 6013 (6.0) 4464 (4.5) 4381 (4.4) 4233 (4.2) 3258 (3.3) 3030 (3.0) 2527 (2.5) 1166 (1.2) 961 (1.0) 864 (0.9) 801 (0.8) 437 0.4)	10 (9.8) 16 (15.7) 13 (12.7) 12 (11.8) 9 (8.8) 3 (2.9) 9 (8.8) 4 (3.9) 3 (2.9) 5 (4.9) 4 (3.9) 3 (2.9) 4 (3.9) 3 (2.9) 1 (1.0) 3 (2.9) 0 (0.0) 0 (0.0)	0.249
ASA-PS, n (%)			
I II III IV V	33,312 (33,3) 56,200 (56.2) 9997 (10.0) 414 (0.4) 68 (0.07)	30 (29.4) 61 (59.8) 11 (10.8) 0 (0.0) 0 (0.0)	0.869
Primary anesthesia provider, n (%)			
Attending anesthesiologist alone Resident + attending anesthesiologist Intern + attending anesthesiologist	15,409 (15.4) 46,263 (46.3) 38,319 (38.3)	6 (5.9) 52 (51.0) 44 (43.1)	0.029
Type of anesthesia, n (%)			
General Volatile General TIVA Regional MAC	77,298 (77.3) 15,776 (15.8) 6524 (6.5) 393 (0.4)	79 (77.5) 21 (20.6) 2 (2.0) 0 (0.0)	0.161
Anesthesia for			
Elective, n (%) Emergency, n (%)	90,794 (90.8) 9197 (9.2)	96 (94.1) 6 (5.9)	0.304
Duration of anesthesia, min (SD)	216 (141)	251 (152)	0.012
Anesthesia on			
Day, n (%) Night, n (%)	96,790 (96.8) 3201 (3.2)	100 (98.0) 2 (2.0)	0.775
Anesthesia on			
Weekday, n (%) Holiday, n (%)	97,977 (98.0) 2014 (2.0)	101 (99.0) 1 (1.0)	0.727

### Predictors of medication errors: firth’s penalized logistic regression

Firth’s penalized logistic regression based on the minimal sufficient adjustment set derived from the DAG identified the primary anesthesia provider as a significant predictor of intraoperative medication errors (Table 4). Compared with cases managed solely by attending anesthesiologists, cases involving trainees were associated with significantly higher odds of error: resident + attending anesthesiologist (OR 2.713; 95% CI, 1.283–6.815; Holm-adjusted *P* = 0.007; FDR q = 0.003) and intern + attending anesthesiologist (OR 3.272; 95% CI, 1.508–8.368; Holm-adjusted *P* = 0.003; FDR q = 0.003) compared with attending anesthesiologist alone. Additionally, surgical cases in the department of otorhinolaryngology had a nominally lower odds of medication error compared with those in obstetrics and gynecology (OR = 0.435; 95% CI 0.187–0.986; unadjusted *P* = 0.046); however, this association did not remain significant after adjusting for multiple comparisons (Holm-adjusted *P* = 0.785; FDR q = 0.444). After multiplicity adjustment, no other covariates—including age, ASA-PS, and surgical urgency—were statistically significant. However, the wide 95% confidence intervals indicate imprecision rather than evidence of no association.

**Table 4:** Multivariable analysis of risk factors for intraoperative medication errors using firth’s penalized logistic regression. P (Holm-adjusted) indicates P values adjusted for the familywise error rate (FWER) using the Holm–Bonferroni method. Q (FDR-adjusted) indicates P values adjusted for the false discovery rate (FDR) using the Benjamini–Hochberg procedure. CI, confidence interval; OR, odds ratio; ASA-PS, American society of anesthesiologists physical status

Variable & Subgroup	Reference	Odds ratio	Odds ratio 95% CI (lower, upper)	*P* unadjusted	*P* Holm-adjusted	q FDR-adjusted
Age	Per 1 year increase	0.999	0.988, 1.012	0.984		
Department of surgery						
Otorhinolaryngology Orthopedic Gastrointestinal Neurosurgery Plastic Urology Pediatric Hepato-Biliary-Pancreatic Vascular Breast and Endocrine Thoracic Cardiac Ophthalmology Dermatology Psychiatry Dental and Oral Other Internal Medicine	Obstetrics and Gynecology	0.435 1.087 1.243 1.204 0.485 1.323 0.963 0.699 1.282 1.097 1.087 1.748 3.232 1.386 3.910 0.496 1.346	0.187, 0.986 0.519, 2.314 0.551, 2.782 0.496, 2.811 0.124, 1.436 0.541, 3.113 0.271, 2.906 0.178, 2.100 0.400, 3.643 0.328, 3.046 0.271, 3.346 0.462, 5.773 0.818, 9.743 0.151, 5.719 0.984, 11.90 0.004, 3.764 0.010, 10.46	0.046 0.824 0.596 0.673 0.203 0.529 0.949 0.547 0.658 0.868 0.894 0.390 0.088 0.717 0.052 0.587 0.844	0.785 1.000 1.000 1.000 1.000 1.000 1.000 1.000 1.000 1.000 1.000 1.000 1.000 1.000 0.837 1.000 1.000	0.444 1.000 1.000 1.000 0.862 1.000 1.000 1.000 1.000 1.000 1.000 1.000 0.497 1.000 0.444 1.000 1.000
ASA-PS						
II III IV V	ASA-PS I	1.117 1.095 1.381 9.321	0.689, 1.840 0.457, 2.415 0.010, 11.82 0.069, 90.38	0.656 0.830 0.835 0.257	1.000 1.000 1.000 1.000	0.835 0.835 0.835 0.835
Anesthesia for emergency	Elective	0.658	0.261, 1.391	0.293		
Primary anesthesia provider						
Resident + attending Intern + attending	Attending anesthesiologist alone	2.713 3.272	1.283, 6.815 1.508, 8.368	0.007 0.002	0.007 0.003	0.007 0.003

### Model fit evaluation

The Firth logistic regression model showed modest discriminative ability (AUC = 0.6472) and a low Brier score (0.00102). Given the rare event rate (0.1%), the Brier score largely reflects accurate prediction of non-events. Calibration was assessed using a bootstrap-corrected calibration plot based on 100 resamples (Supplementary Fig. 2). The bias-corrected curve showed a modest deviation from the ideal line, suggesting minor over- or underestimation of predicted probabilities across risk strata.

### Sensitivity analyses

Sensitivity analyses using a full covariate model and an alternative minimal adjustment set supported the robustness of the primary results. Across all models, the category of primary anesthesia provider remained significantly associated with medication errors. Specifically, cases involving trainees—particularly when multiple anesthesia providers were involved—showed consistently higher odds of error compared with those managed solely by attending anesthesiologists (Supplementary Table 2). These findings suggest that provider-related factors, including level of experience and complexity of team composition, contribute to the occurrence of medication errors.

## Discussion

Our study yielded two primary findings on intraoperative medication errors. First, the overall incidence was low (approximately 0.1%), and most errors were mild to moderate in severity, with no permanent patient harm. Second, the only statistically significant independent predictor was anesthesia provider experience. Cases managed by residents with attending or interns with attending supervision had markedly higher error risks than those managed by attending anesthesiologists.

In this large-scale, single-center retrospective study, we identified intraoperative medication errors in 0.10% of surgical cases managed by the anesthesia department. Although this incidence is lower than those reported in previous international studies (ranging from 0.37% to 0.75%) [2, 5, 6, 15], it is closer to the rate found in a prior 15-year analysis from Japan (0.078%) [14], possibly reflecting similar reporting practices. These discrepancies may be attributed to differences in study design, definitions of medication errors, and reporting methodologies. Notably, studies utilizing direct observation or real-time reporting tend to report higher error rates compared with retrospective analyses [13, 20]. While self-reporting may introduce bias, our institution employs multiple reporting pathways—including direct reports by interns, residents, and attendings, as well as supervisor and nurse-initiated reports—which likely reduce underreporting. Dual attending anesthesiologist verification was applied to ensure that only genuine intraoperative medication errors meeting predefined criteria were analyzed. This process led to the exclusion of five reports (4.7%) judged as outside the intraoperative context or near-miss events, confirming that this verification step was appropriate and effective in maintaining data validity.

Regarding error types, incorrect dosing, substitution, and omission errors were the most prevalent with antibiotics, remifentanil, and opioids frequently implicated. These findings align with recent systematic reviews and meta-analyses. These reports indicate that substitution and dosing errors are the most prevalent during anesthesia, and that antibiotics, opioids, and muscle relaxants are the drug classes most often involved [2, 21, 22]. Omission errors, especially with remifentanil and antibiotics, likely reflect the complexity of perioperative medication management and the necessity for precise timing and continuous administration.

Our multivariable analysis identified provider-related factors as significantly associated with the risk of medication errors. Compared with cases managed solely by attending anesthesiologists, those involving interns or residents exhibited approximately threefold higher odds of error occurrence. This aligns with earlier reports [2, 13, 14] suggesting that trainees are more prone to lapses in vigilance, dosing miscalculations, or unfamiliarity with protocols, particularly under time pressure. Importantly, all severe errors (Level 3b) occurred in cases managed by residents, underscoring the need for robust supervisory frameworks, especially in complex surgical settings such as cardiac or neurosurgical anesthesia. As shown in Table 2, residents accounted for a greater proportion of reported errors, but the majority of events across all provider levels were mild (Level 1–2). Although Level 3b events appeared more frequently in resident-managed cases, this observation should be interpreted cautiously in light of case acuity. In this study, high-acuity cases were defined as those meeting at least one of the following criteria: (1) surgical specialty in cardiac, pediatric, neurosurgical, thoracic, or vascular surgery; (2) emergency surgery; or (3) ASA physical status ≥ 3. Based on this definition, the proportion of high-acuity cases was 36.6% for attending-only, 51.4% for resident + attending, and 8.7% for intern + attending cases. These data suggest that residents were more frequently involved in high-acuity surgeries, which may have contributed to the higher number of severe events observed in this group. However, because this study was not designed to adjust for case complexity, the association between training level and error severity should be interpreted with caution.

Prior literature provides important theoretical support for our finding that medication errors were more common when care involved residents or interns. Supervision quality in anesthesiology has been shown to be a measurable construct with strong validity and reliability, as demonstrated by the development and psychometric evaluation of a nine-item supervision assessment instrument [23]. Moreover, inadequate or low-quality supervision has repeatedly been associated with higher frequencies of self-reported medical errors among anesthesiology trainees in national surveys, indicating that insufficient oversight can adversely affect patient safety [24]. These studies suggest that supervision quality varies significantly in routine practice and is not easily apparent without systematic measurement. Taken together, this body of evidence strengthens the plausibility and generalizability of our results, underscoring that targeted improvements in supervisory practices may represent a modifiable strategy for reducing intraoperative medication errors.

Notably, most perioperative/procedural factors—including patient age, surgical urgency, and ASA physical status—were not statistically associated with medication-error risk after multivariable adjustment. This accords with our hypothesis and prior work [5], suggesting human factors (provider experience, communication, team dynamics) may outweigh situational variables in intraoperative drug safety. The only nominal association was observed for surgical department: otorhinolaryngology showed a lower odds of medication error compared with obstetrics and gynecology (OR = 0.435; 95% CI 0.187–0.986; unadjusted *P* = 0.046), but this difference was not statistically significant after adjustment for multiple comparisons. Possible reasons include more standardized workflows, shorter anesthesia duration, or differences in team structure and patient risk. These interpretations remain tentative given the observational design and warrant further study. Although age, ASA-PS, and surgical urgency were not statistically significant after multiplicity adjustment, the broad 95% confidence intervals preclude definitive conclusions of no effect.

Systemic contributors include communication failures [25], time pressure [26], and provider fatigue [27]; the complexity of anesthesia work and medication mishandling further drive anesthesia-related errors [21, 28]. A multicenter study showed that each anesthesia handoff increased major morbidity by 8% (OR 1.08), underscoring the need for standardized handoffs [29]. In our cohort, 10 (9.8%) intraoperative errors were linked to communication failures, often during care transitions. Mitigation strategies include double-checking high-risk drugs, prefilled syringes or barcode verification [30], and fostering open communication; standardized handoffs and simulation-based team training may further reduce transition-related risk [29].

Decades of research support a systems-based approach to medication safety, which focuses on redesigning workflows and environments to account for human limitations, rather than relying on vigilance or attributing blame. Webster emphasizes that exhortation and individual-focused strategies are weak mechanisms for lasting safety improvement, especially in high-risk settings such as anesthesia [31]. In line with this, Maximous et al. demonstrated that multimodal interventions—such as barcode scanning, standardized labeling, and drug cart redesign—reduced anesthesia medication errors by up to 41% [32]. These data suggest that the increased error risk observed among trainees may reflect insufficient system safeguards during supervision, transitions, or task handoffs. In addition to robust supervision, incorporating system-level interventions—such as simulation-based training and enhanced drug identification systems—may further improve medication safety by reducing reliance on individual vigilance.

This study has several strengths. First, it is based on a large dataset of over 100,000 anesthesia-managed surgical cases collected over more than a decade at a high-volume academic institution. This sample size enhances statistical power and reliability, particularly given the rare incidence of intraoperative medication errors. Second, the study used Firth’s penalized logistic regression to handle rare outcome events and reduce bias from data separation. Third, the analysis was guided by a prespecified DAG, grounding covariate selection in causal inference rather than data-driven modeling alone. Furthermore, sensitivity analyses using an alternative minimal adjustment set confirmed the robustness of the primary findings. Lastly, the study leveraged routine electronic anesthesia records and institutional incident-reporting systems, enhancing generalizability to similar settings. Together, these strengths support internal validity and provide important insights into provider-related risk factors for medication errors in the operating room.

This study has several limitations. First, its retrospective, single-center design may have introduced under-ascertainment and outcome misclassification, given reliance on self-reported medication-error documentation. To mitigate under-reporting, our institution uses multiple reporting pathways—direct submissions via the institutional incident-reporting system and the intraoperative anesthesia information system, plus supplementary reports from supervising physicians and perioperative nurses—which may have improved case ascertainment. Second, we did not assess intermediate or long-term clinical outcomes (e.g., postoperative complications, readmissions, functional recovery), which could be affected by medication errors but were not captured in our dataset. Third, because this was a single Japanese academic center with a specific anesthesia training structure and a relatively low proportion of high-acuity/emergency cases, generalizability is limited. Fourth, despite adjusting for key confounders using a DAG-based approach, supervision intensity, intraoperative handoffs, and provider workload were not captured with sufficient granularity; therefore, residual confounding may persist. Fifth, the frequency of intraoperative handoffs could not be quantified because the electronic anesthesia record system does not retain a continuous record of provider changes, and brief relief periods are common in daily practice. Consequently, care transitions—including intraoperative handoffs and other communication failures—could only be qualitatively assessed. Contributory factors were derived from free-text descriptions in the institutional incident-reporting system, which were completed by the reporting anesthesia providers. These narratives often contained contextual details such as communication breakdowns, workflow interruptions, or knowledge-based errors. However, because these data were based on unstructured provider narratives rather than time-stamped user logs or standardized root cause analyses, their identification may be incomplete or subjective. Finally, missing data were minimal (< 0.01%) and handled via complete-case analysis; given the extent of missingness, material bias is unlikely, although unverifiable assumptions about the missing-data mechanism remain. Because medication errors were rare, summary performance metrics should be interpreted cautiously, and our models are not intended for individual risk prediction. Overall, these limitations suggest that our inferences should be interpreted as associations rather than causal effects, and external validation in other settings with different case-mix and supervision structures is warranted.

## Conclusions

In this large single-center study, the rate of intraoperative medication errors was 0.1% and was primarily associated with anesthesia provider experience and team composition. These findings suggest the need for targeted training, robust supervision, and system-level safeguards—such as standardized handoff protocols and decision support tools—to prevent medication-related harm in the operating room. Future prospective research and multicenter collaborations are warranted to validate our results and explore effective interventions to enhance medication safety in anesthesia practice.

## Supplementary Information

Supplementary Material 1: Supplementary table 1.

Supplementary Material 2: Supplementary figure 1A. Initial DAG (before adjustment). The exposure variable is the primary anesthesia provider , and the outcome is intraoperative medication error. Blue nodes indicate ancestors of the outcome, and pink nodes indicate ancestors of both the exposure and the outcome. Causal paths are shown with green arrows, and biasing paths with pink arrows. This DAG displays all considered variables and potential confounding pathways prior to adjustment set selection. DAG, directed acyclic graph.

Supplementary Material 3: Supplementary figure 1B. Minimal adjustment set 1. DAG showing the minimal sufficient adjustment set for estimating the total effect of primary anesthesia provider on medication errors, comprising age, ASA-PS classification, surgical department, and surgical urgency (elective/emergency). Confounders included in the minimal set are highlighted. DAG, directed acyclic graph; ASA-PS, American Society of Anesthesiologists Physical Status.

Supplementary Material 4: Supplementary figure 1C. Minimal adjustment set 2. DAG representing the alternative minimal sufficient adjustment set identified by DAGitty, comprising age, surgical department, surgical urgency (elective/emergency), and type of anesthesia. This set was used for sensitivity analyses to evaluate the robustness of the primary findings. DAG, directed acyclic graph.

Supplementary Material 5: Supplementary table 2.

Supplementary Material 6: Supplementary figure 2. Calibration plot of the logistic regression model predicting intraoperative medication errors. The x-axis shows the predicted probabilities, while the y-axis represents the observed probabilities. The solid black line (“Apparent”) reflects the model's calibration on the original dataset. The dotted grey line (“Ideal”) represents perfect calibration, where predicted and observed probabilities are equal. The bias-corrected line (“Bias-corrected”) was generated using 100 bootstrap resamples to adjust for overfitting and reflects the model’s expected performance on new data.

Supplementary Material 7: Supplementary table 3.

## Data Availability

The datasets generated and analyzed during the current study are not publicly available due to institutional policy but are available from the corresponding author on reasonable request.
