# Supplementary material for: Primary anesthesia provider characteristics and risk factors for intraoperative medication errors: a retrospective cohort study
Source: BMC Anesthesiol. 2025 Dec 13;26:44. doi: 10.1186/s12871-025-03539-4 (PMC12817502; doi:10.1186/s12871-025-03539-4)
Supplement: Supplementary file 1 — Supplementary Material 1: Supplementary table 1. [file 12871_2025_3539_MOESM1_ESM.docx]

| Level | Persistence of harm | Severity of harm | Description |
| --- | --- | --- | --- |
| Level 0 | None | None | An error or malfunction in drugs or medical devices was identified, but it was not actually applied to the patient. |
| Level 1 | None | None | No actual harm to the patient, although a potential influence cannot be completely ruled out. |
| Level 2 | Transient | Mild | No treatment was administered. However, enhanced monitoring of the patient, mild changes in vital signs, or tests to ensure safety may have been required. |
| Level 3a | Transient | Moderate | Simple treatment was required (e.g., disinfection, application of a compress, suturing, or administration of painkillers). |
| Level 3b | Transient | Severe | Intensive treatment was required (e.g., significant changes in vital signs, use of mechanical ventilation, surgery, extended hospitalization, inpatient admission from outpatient care, or fractures). |
| Level 4a | Permanent | Mild to moderate | A permanent disability or sequelae occurred, but without significant functional impairment or cosmetic concern. |
| Level 4b | Permanent | Moderate to severe | A permanent disability or sequelae occurred with significant functional impairment or cosmetic disfigurement. |
| Level 5 | Death | — | Death occurred (excluding natural progression of the primary disease). |

**Supplementary table 1.** Classification of incident severity approved at the 1st General Meeting of the Medical Safety Management Committee of the National University Hospital Council of Japan. For further details, see: <https://nuhc.jp/wp-content/themes/NUHC/Portals/0/images/activity/report/sgst_category/safety/incidentcategory.pdf>.
