# Supplementary figures and images for: Primary anesthesia provider characteristics and risk factors for intraoperative medication errors: a retrospective cohort study
Source: BMC Anesthesiol. 2025 Dec 13;26:44. doi: 10.1186/s12871-025-03539-4 (PMC12817502; doi:10.1186/s12871-025-03539-4)

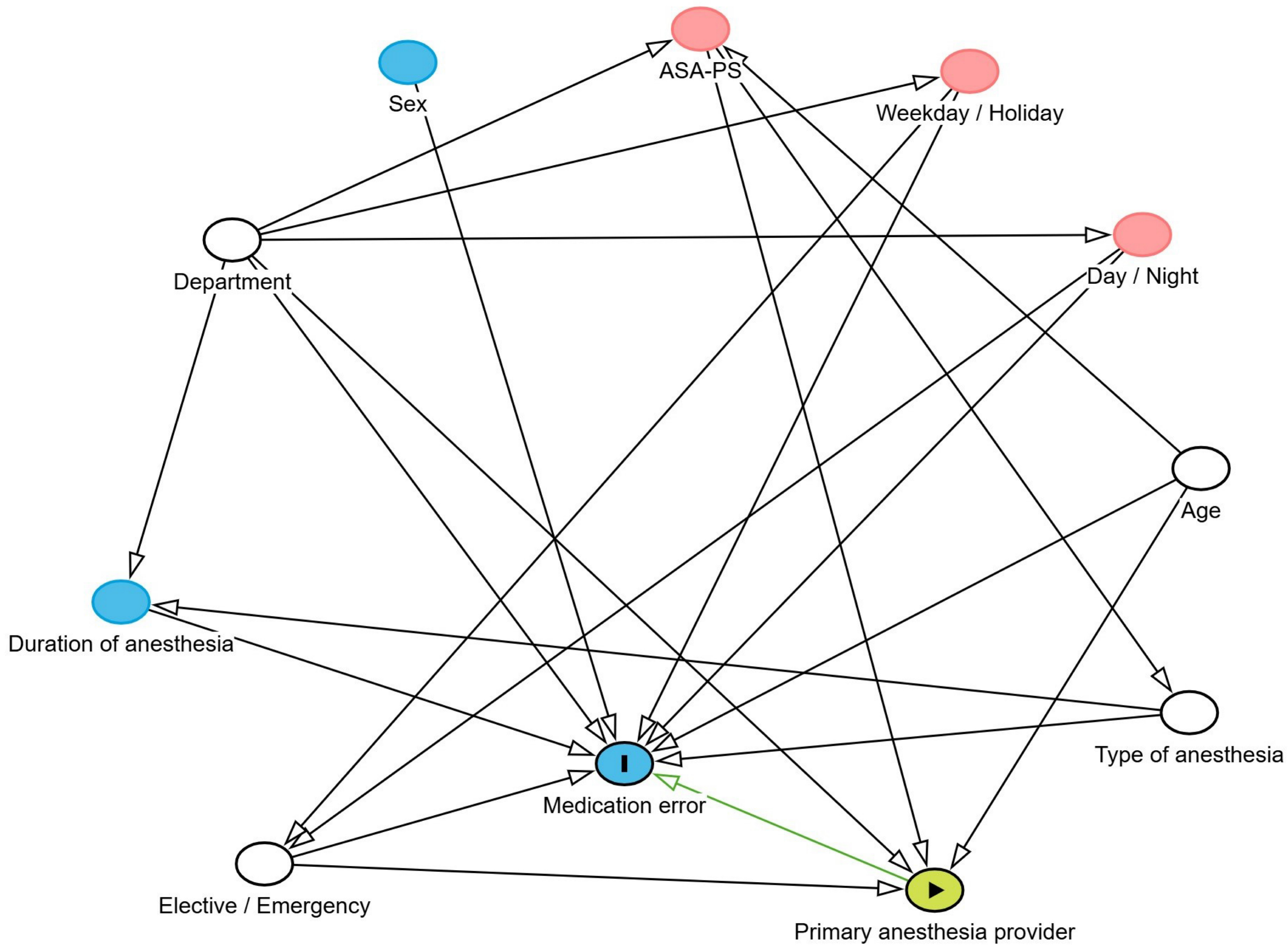

Supplement: Supplementary file 3 — Supplementary Material 3: Supplementary figure 1B. Minimal adjustment set 1. DAG showing the minimal sufficient adjustment set for estimating the total effect of primary anesthesia provider on medication errors, comprising age, ASA-PS classification, surgical department, and surgical urgency (elective/emergency). Confounders included in the minimal set are highlighted. DAG, directed acyclic graph; ASA-PS, American Society of Anesthesiologists Physical Status. [file 12871_2025_3539_MOESM3_ESM.pdf]

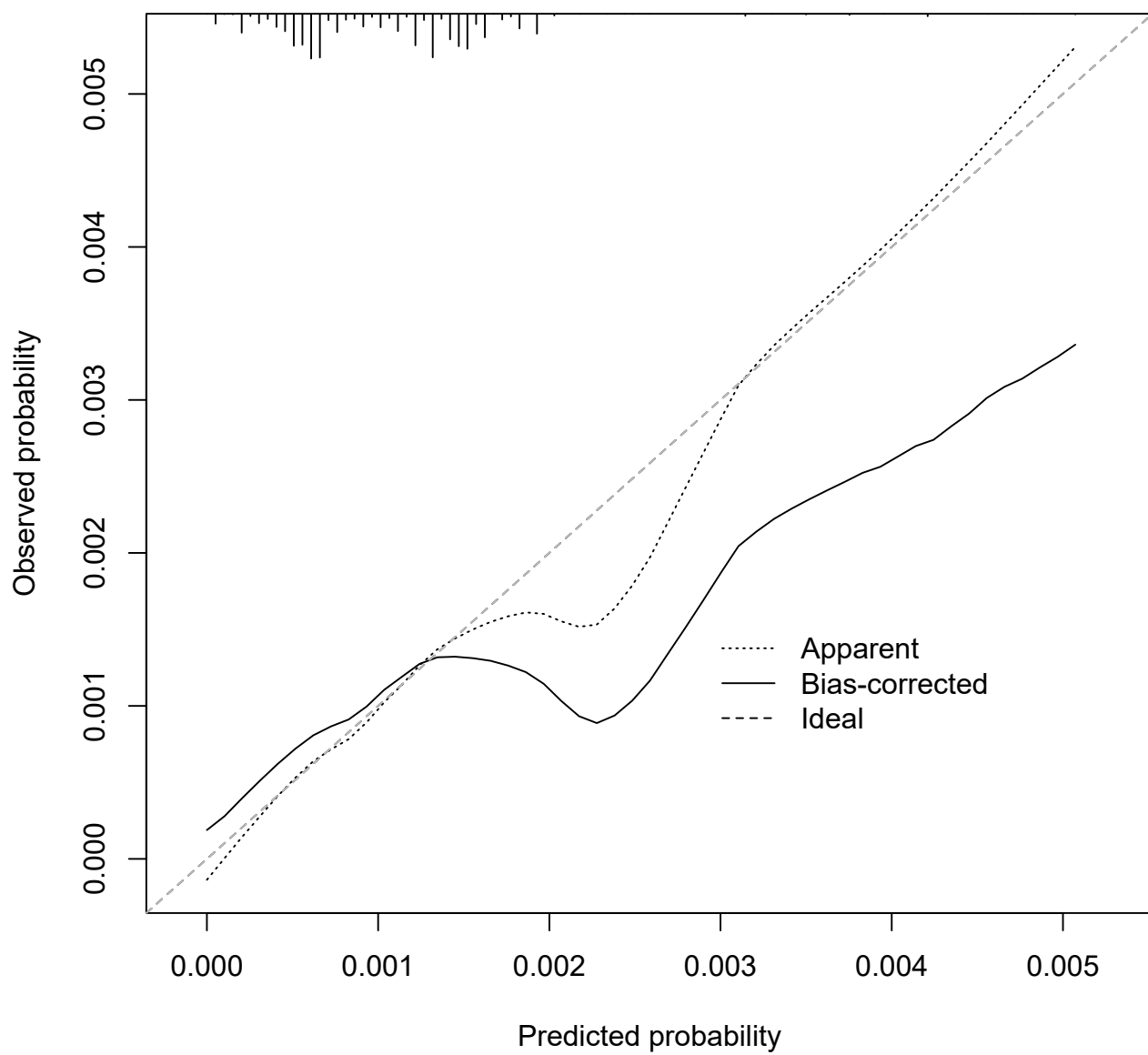

Supplement: Supplementary file 6 — Supplementary Material 6: Supplementary figure 2. Calibration plot of the logistic regression model predicting intraoperative medication errors. The x-axis shows the predicted probabilities, while the y-axis represents the observed probabilities. The solid black line (“Apparent”) reflects the model's calibration on the original dataset. The dotted grey line (“Ideal”) represents perfect calibration, where predicted and observed probabilities are equal. The bias-corrected line (“Bias-corrected”) was generated using 100 bootstrap resamples to adjust for overfitting and reflects the model’s expected performance on new data. [file 12871_2025_3539_MOESM6_ESM.pdf]
