# Supplementary material for: Primary anesthesia provider characteristics and risk factors for intraoperative medication errors: a retrospective cohort study
Source: BMC Anesthesiol. 2025 Dec 13;26:44. doi: 10.1186/s12871-025-03539-4 (PMC12817502; doi:10.1186/s12871-025-03539-4)
Supplement: Supplementary file 5 — Supplementary Material 5: Supplementary table 2. [file 12871_2025_3539_MOESM5_ESM.docx]

**Supplementary table 2. Sensitivity analyses of predictors for intraoperative medication errors using Firth’s penalized logistic regression.** Comparison of two alternative models: (1) a full model including all available covariates; and (2) an alternative minimal adjustment set derived from a secondary DAG (age, surgical department, surgical urgency, and type of anesthesia; supplementary figure 2C). P (Holm-adjusted) indicates P values adjusted for the familywise error rate (FWER) using the Holm–Bonferroni method. q (FDR-adjusted) indicates P values adjusted for the false discovery rate (FDR) using the Benjamini–Hochberg procedure. Odds ratios (ORs), 95% confidence intervals (CIs), and P values are shown. DAG, directed acyclic graph; ASA-PS, American Society of Anesthesiologists Physical Status; TIVA, total intravenous anesthesia; MAC, monitored anesthesia care.

| Variables | Reference | Full Firth model | | | | Alternative DAG-based minimal Firth model | | | |
| --- | --- | --- | --- | --- | --- | --- | --- | --- | --- |
|  |  | OR (95% CI) | P  unadjusted | P  Holm-adjusted | q  FDR-adjusted | OR (95% CI) | P  unadjusted | P  Holm-adjusted | q  FDR-adjusted |
| Age | Per 1 year increase | 0.998 (0.987,1.010 ) | 0.810 |  |  | 1.001 (0.990, 1.012) | 0.872 |  |  |
| Sex, male | Female | 0.783 (0.503, 1.225) | 0.282 |  |  |  |  |  |  |
| Department of Surgery  Otorhinolaryngology  Orthopedic  Gastrointestinal  Neurosurgery  Plastic  Urology  Pediatric  Hepato-Biliary-Pancreatic  Vascular  Breast and Endocrine  Thoracic  Cardiac  Ophthalmology  Dermatology  Psychiatry  Dental and Oral  Other Internal Medicine | Obstetrics and Gynecology | 0.457 (0.187, 1.086)  1.083 (0.505, 2.355)  1.221 (0.511, 2.885)  1.018 (0.403, 2.487)  0.494 (0.124, 1.512)  1.405 (0.539, 3.531)  1.011 (0.269, 3.278)  0.669 (0.164, 2.105)  1.264 (0.374, 3.839)  0.979 (0.292, 2.724)  0.963 (0.232, 3.130)  1.269 (0.317, 4.497)  3.445 (0.841, 10.97)  1.486 (0.160, 6.312)  3.899 (0.898, 13.64)  0.524 (0.004, 4.083)  1.523 (0.012, 12.36) | 0.076  0.837  0.649  0.968  0.229  0.478  0.986  0.512  0.691  0.970  0.954  0.723  0.081  0.667  0.067  0.622  0.786 | 1.000  1.000  1.000  1.000  1.000  1.000  1.000  1.000  1.000  1.000  1.000  1.000  1.000  1.000  1.000  1.000  1.000 | 0.458  0.978  0.978  0.978  0.974  0.978  0.978  0.978  0.978  0.978  0.978  0.978  0.978  0.978  0.458  0.978  0.978 | 0.376 (0.161, 0.862)  0.972 (0.462, 2.080)  1.112 (0.486, 2.530)  1.016 (0.413, 2.412)  0.413 (0.105, 1.241)  1.231 (0.500, 2.922)  0.817 (0.225, 2.539)  0.610 (0.154, 1.856)  1.124 (0.351, 3.200)  0.927 (0.277, 2.571)  0.842 (0.208, 2.632)  1.345 (0.393, 3.854)  2.715 (0.678, 8.349)  1.236 (0.134, 5.131)  2.827 (0.687, 9.164)  0.428 (0.003, 3.261)  1.160 (0.009, 9.037) | 0.021  0.942  0.799  0.971  0.120  0.642  0.737  0.403  0.834  0.890  0.782  0.610  0.143  0.812  0.137  0.501  0.920 | 0.359  1.000  1.000  1.000  1.000  1.000  1.000  1.000  1.000  1.000  1.000  1.000  1.000  1.000  1.000  1.000  1.000 | 0.359  0.971  0.971  0.971  0.608  0.971  0.971  0.971  0.971  0.971  0.971  0.971  0.608  0.971  0.608  0.971  0.971 |
| ASA-PS  II  III  IV  V | ASA-PS I | 1.177 (0.724, 1.945)  1.169 (0.487, 2.582)  1.358 (0.010, 11.69)  8.815 (0.065, 87.37) | 0.513  0.714  0.843  0.267 | 1.000  1.000  1.000  1.000 | 0.843  0.843  0.843  0.843 |  |  |  |  |
| Primary anesthesia provider  Resident + attending  Intern + attending | Attending anesthesiologist  alone | 2.664 (1.254, 6.716)  3.193 (1.463, 8.199) | 0.009  0.002 | 0.009  0.005 | 0.009  0.005 | 2.758 (1.304, 6.933)  3.178 (1.461, 8.134) | 0.006  0.002 | 0.006  0.005 | 0.006  0.005 |
| Type of anesthesia  TIVA  Regional  MAC | Volatile | 1.163 (0.656, 1.969)  0.369 (0.073, 1.166)  1.658 (0.013, 12.34) | 0.593  0.096  0.745 | 1.000  0.287  0.287 | 0.745  0.287  0.745 | 1.209 (0.683, 2.042)  0.338 (0.068, 1.029)  1.328 (0.010, 9.692) | 0.501  0.057  0.850 | 1.000  0.172  1.000 | 0.751  0.172  0.850 |
| Anesthesia for emergency | Elective | 0.956 (0.321, 2.283) | 0.927 |  |  | 0.730 (0.291, 1.536) | 0.433 |  |  |
| Duration of anesthesia | Per 1 min increase | 1.001 (0.999, 1.002) | 0.163 |  |  |  |  |  |  |
| Anesthesia on night | Day | 1.004 (0.183, 3.855) | 0.996 |  |  |  |  |  |  |
| Anesthesia on holiday | Weekday | 0.833 (0.087, 3.834) | 0.836 |  |  |  |  |  |  |
