# Supplementary material for: Primary anesthesia provider characteristics and risk factors for intraoperative medication errors: a retrospective cohort study
Source: BMC Anesthesiol. 2025 Dec 13;26:44. doi: 10.1186/s12871-025-03539-4 (PMC12817502; doi:10.1186/s12871-025-03539-4)
Supplement: Supplementary file 7 — Supplementary Material 7: Supplementary table 3. [file 12871_2025_3539_MOESM7_ESM.docx]

**Supplementary table 3.** Clinical characteristics and courses of patients involved in level 3b medication error incidents. This table summarizes detailed case information for patients who experienced level 3b intraoperative medication errors, classified as events causing temporary harm requiring intervention. For each case, patient demographics, surgical and anesthetic details, type of medication error, responsible provider, and postoperative course are presented. ASA-PS, American Society of Anesthesiologists Physical Status; TIVA, total intravenous anesthesia; MAC, monitored anesthesia care; ICU, intensive care unit; PTC, post-tetanic count.

| case | Patient characteristics | Surgery and anesthesia | Medication error | Clinical course |
| --- | --- | --- | --- | --- |
| 1 | Sex: female  ASA-PS: II | Department: Obstetrics and Gynecology  Procedure: cesarean delivery  Anesthesia: regional  Operation time: 66min  Anesthesia time : 101min | Type of error: incorrect dose  Provider: resident  An overdose of 1.5mg morphine hydrochloride was administered intrathecally | Naloxone was administered postoperatively, and the patient was monitored overnight in the ICU. |
| 2 | Sex: male  ASA-PS: III | Department: Cardiac surgery  Procedure: mitral valvuloplasty  Anesthesia: TIVA  Operation time: 244min  Anesthesia time : 346min | Type of error: substitution  Provider: resident  0.9mg of adrenaline was administered intravenously instead of heparin | Systolic blood pressure temporarily increased to 300 mmHg, which was promptly normalized with vasodilator administration. |
| 3 | Sex: male  ASA-PS: III | Department: Neurosurgery  Procedure: endoscopic hematoma evacuation  Anesthesia: Volatile  Operation time: 68min  Anesthesia time : 123min | Type of error: incorrect dose  Provider: resident  An overdose of rocuronium (1.1mg/kg) was administered to a patient with spinal and bulbar muscular atrophy in anesthesia induction | PTC remained at 0 until the end of surgery. The patient was transferred to the ICU without extubation. Several hours later, after confirming the presence of PTC, sugammadex was administered and the patient was extubated. |
| 4 | Sex: male  ASA-PS: II | Department: Vascular surgery  Procedure: thoracic endovascular aortic repair  Anesthesia: Volatile  Operation time: 170min  Anesthesia time : 228min | Type of error: incorrect dose  Provider: resident  Lidocaine was administered intravenously at 10 times the normal rate (500 mg/h) | The ICU physician noticed a lidocaine overdose, postoperatively. In the ICU, the patient was agitated for two hours, unresponsive to communication, and required physical restraints on the limbs. |
